# Neglected Major Causes of Death Much Deadlier Than COVID-19

**DOI:** 10.34172/jrhs.2020.13

**Published:** 2020-05-17

**Authors:** Jalal Poorolajal

**Affiliations:** ^1^Department of Epidemiology, School of Public Health, Hamadan University of Medical Sciences, Hamadan, Iran; ^2^Modeling of Noncommunicable Diseases Research Center, School of Public Health, Hamadan University of Medical Sciences, Hamadan, Iran

COVID-19, the disease caused by the new coronavirus, was first detected as pneumonia of unknown cause in Wuhan, China on December 31, 2019^1^. In a very short time, the outbreak was declared a public health emergency worldwide by the World Health Organization on January 30, 2020 and then characterized as a pandemic on March 11. By May 15, 2020, over 4.5 million people worldwide were infected by the new coronavirus and 304,127 people died from the disease^2^.

Although COVID-19 is a highly contagious and dangerous infectious disease, several deadlier diseases are killing thousands of people worldwide every day, but unfortunately, many people do not pay attention to them. Now the critical question is why people do not pay attention to these major causes of death as much as they do to COVID-19.

It may be shocking to many people if they hear that more than 49,041 people died due to cardiovascular diseases, 26,301 people due to cancer, 20,822 people due to hypertension, and 19,178 people from air pollution within the past 24 hours worldwide. It may be unbelievable for many people if they find out that the same number of death from the same diseases have been occurring for several years and will occur in the following days, weeks, months, and years! Probably, a majority of people may not believe these figures. Unfortunately, not only these figures are true but also the situation is much more devastating than this. According to the report of the World Health Organization in 2018^3^ and other sources^4,5^, apparently, millions of people died of 19 major causes of death worldwide (Table 1).

**Table 1:** Major causes of death worldwide based on the World Health Organization report^3^ and other sources^4,5^

**Causes**	**Year**	**Death**
Cardiovascular diseases	2018	17,900,000
Cancer	2018	9,600,000
Hypertension	2018	7,500,000
Air pollution	2018	7,000,000
Chronic obstructive respiratory diseases	2018	3,170,000
Alcohol	2018	3,000,000
Pneumonia	2019	2,560,000
Diabetes	2018	1,600,000
Tuberculosis	2018	1,500,000
Road traffic accidents	2018	1,350,000
Hepatitis B and C	2018	1,340,000
Tobacco	2018	1,200,000
Suicide	2018	800,000
HIV/AIDS	2018	770,000
Influenza	2018	650,000
Diarrhea	2018	525,000
Homicides	2018	470,000
Malaria	2018	405,000

**Figure 1:**
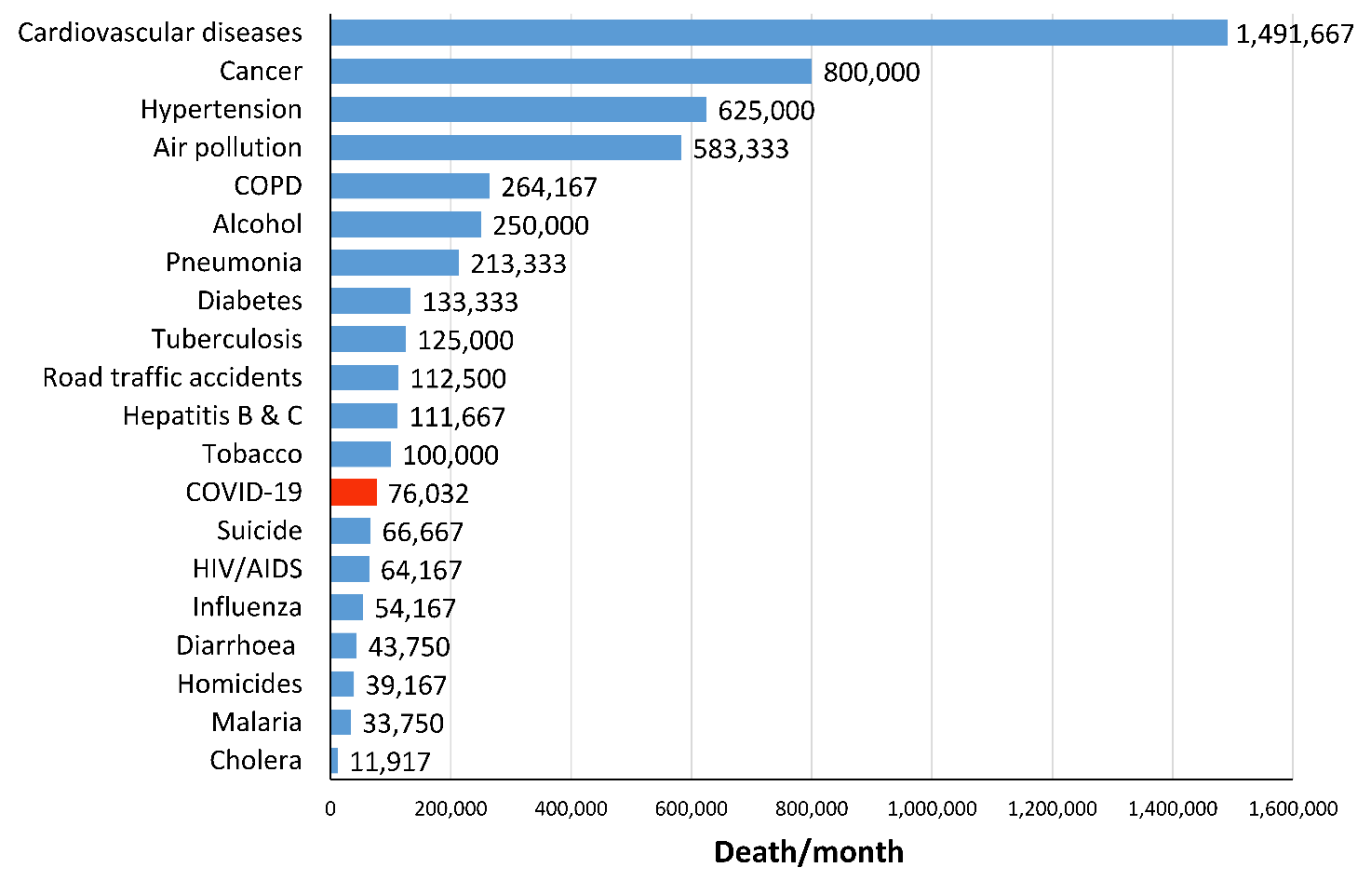
The death rate of 20 leading causes of death worldwide based on the World Health Organization report in 2018^3^ and other sources^4,5^ [To obtain death rate per month, the annual deaths due to COVID-19 were divided by 4 and that of other diseases were divided by 12.]

The depth of the tragedy is shown in Figure 1. This figure shows the most common causes of death worldwide. The rank of COVID-19 is 13^th^ among 20 major causes of death. That means, based on current evidence, a diverse group of diseases and health-related conditions kill much many more people every day than COVID-19 does worldwide. Of course, changing upward or downward in the rank of COVID-19 over time is possible depending on whether the disease progresses or regresses. Furthermore, the incidence rate and death rate of COVID-19 are affected by the validity and availability of diagnostic tests. Accordingly, many expert believe that more people have died from coronavirus than reported.

Now we return to the critical question: "why do people not pay attention to these major causes of death as much as they do to COVID-19?" COVID-19 is new pneumonia associated with huge health and economic crises. We are now in the middle of crises and Covid-19 is still the hot topic of worldwide news during the past four months. An international campaign has been launched to curb the disease. Unfortunately, despite the efforts made so far, COVID-19 seems to be around for a long time. In that case, people will never tolerate the restrictions adopted to reduce the spread of the disease for a long time. As a result, the recommended preventive measures, such as stay-at-home, physical distancing, and lockdown restrictions, will be eased gradually and life will inevitably return to normal while the disease still kills many people every day. We will forget the catastrophic consequences of this horrible pandemic very soon as we did heretofore other much deadlier diseases. The reality is that we are human. This is the inherent characteristic of human that get used to any situation and phenomenon that takes for a long time particularly when there is no easy solution for it.

The transmission of COVID-19 is faster than many contagious diseases we have ever known. This issue caused great panic among people worldwide. Paying special attention to the disease and taking preventive measures to curb and control the disease is a health priority. This paper does not intend to underestimate the importance of COVID-19, rather it reminds the importance of other dangerous but neglected causes of death that kill thousands of people worldwide every day. Unfortunately, people hear little about the importance and catastrophic consequences of these common causes of death in the media. Therefore, they do not pay enough attention to them while a majority of the deaths due to the aforementioned diseases will be prevented by changing lifestyles and improving health services. COVID-19 reminded us how vulnerable we are and how forgetful and unaware of major causes of death we are!
